# Root Trait Variation in Lentil (*Lens culinaris* Medikus) Germplasm under Drought Stress

**DOI:** 10.3390/plants10112410

**Published:** 2021-11-09

**Authors:** Swati Priya, Ruchi Bansal, Gaurav Kumar, Harsh Kumar Dikshit, Jyoti Kumari, Rakesh Pandey, Amit Kumar Singh, Kuldeep Tripathi, Narender Singh, N. K. Prasanna Kumari, Shiv Kumar, Ashok Kumar

**Affiliations:** 1 Division of Germplasm Evaluation, ICAR-National Bureau of Plant Genetic Resources, New Delhi 110012, India; swatipriya9013@gmail.com (S.P.); kumgaurav66@gmail.com (G.K.); jyoti.kumari@icar.gov.in (J.K.); kuldeep.tripathi@icar.gov.in (K.T.); Ashok.Kumar28@icar.gov.in (A.K.); 2 Department of Botany, Kurukshetra University, Haryana 136119, India; nsheorankuk@yahoo.co.in; 3 Division of Genetics, ICAR-Indian Agricultural Research Institute, New Delhi 110012, India; 4 Division of Plant Physiology, ICAR-Indian Agricultural Research Institute, New Delhi 110012, India; Rakesh.Pandey@icar.gov.in; 5 Division of Genomic Resources, ICAR-National Bureau of Plant Genetic Resources, New Delhi 110012, India; amit.singh5@icar.gov.in; 6 CSIR-National Institute of Science Communication and Information Resources, New Delhi 110012, India; prasanna.bhu@gmail.com; 7 International Centre for Agricultural Research in Dryland Areas, Avenue Hafiane Cherkaoui, Rabat 10112, Morocco; sk.agrawal@cgiar.org

**Keywords:** drought, germplasm, lentil, phenotyping, root traits, variability

## Abstract

Drought is the most critical environmental factor across the continents affecting food security. Roots are the prime organs for water and nutrient uptake. Fine tuning between water uptake, efficient use and loss determines the genotypic response to water limitations. Targeted breeding for root system architecture needs to be explored to improve water use efficiency in legumes. Hence, the present study was designed to explore root system architecture in lentil germplasm in response to drought. A set of 119 lentil (*Lens culinaris* Medik.) genotypes was screened in controlled conditions to assess the variability in root traits in relation to drought tolerance at seedling stage. We reported significant variation for different root traits in lentil germplasm. Total root length, surface area, root volume and root diameter were correlated to the survival and growth under drought. Among the studied genotypes, the stress tolerance index varied 0.19–1.0 for survival and 0.09–0.90 for biomass. Based on seedling survival and biomass under control and drought conditions, 11 drought tolerant genotypes were identified, which may be investigated further at a physiological and molecular level for the identification of the genes involved in drought tolerance. Identified lines may also be utilised in a lentil breeding program.

## 1. Introduction

Drought is the most critical environmental factor limiting crop productivity worldwide. It is becoming recurrent and threatening to the global food supply [1,2]. Drought may worsen in the future, affecting two-thirds of the global population [3]. The genetic improvement of crops for drought tolerance is the most sustainable and economical solution ensuring food security in unpredictable climatic conditions.

Water limitation leads to a series of changes at the morphological, physiological, biochemical and molecular level in plants, disturbing growth and development. The plant’s response depends on the growth stage, genetic potential, stress intensity and duration [4]. However, the seedling and reproductive stages are extremely sensitive to drought and may impact the yield significantly [5]. Disturbed water relations, poor germination and seedling vigour, decline in carbon fixation and assimilate partitioning, reduced pollen fertility, grain filling and low sink activity were reported under stress conditions [6,7,8]. Breeding for water-use efficiency and root traits is the economical approach to manage water-limited environments. Though selection for water-use efficiency has not gained much attention by breeders in legumes, selection for yield traits under stress may be indirectly associated with the desired root traits for the target environment [9]. In recent years, studies in legumes have shown the correlation between root system architecture (RSA) in terms of primary root length (PRL) and yield under drought conditions in soybean [10], common bean [11], chickpea [12] and lentil [6].

The lentil is grown widely throughout the world, including the Indian sub-continent, the Middle East, Northern and East Africa, Southern Europe, North and South America, Australia and Western Asia. Besides fixing atmospheric nitrogen, lentil seeds are a rich source of protein, dietary fibre, vitamins and antioxidants [13]. The crop is cultivated on conserved/residual moisture and experiences intermittent drought in South Asia. Studies have been conducted for the identification of drought tolerant lines in cultivated and wild lentil [14,15]. Drought response deployed by different lentil species (*Lens orientalis*, *Lens tomentosus*, *Lens odemensis*, *Lens lamottei*, *Lens ervoides* and *Lens culinaris*) belonging to the same gene pool were different among each other [15]. The results showed that the drought response of different species did not reflect their genetic similarities. In cultivated lentil, a shallow root system and a deep root system in wild lentil was reported under stress, given only a few genotypes were taken into consideration. To explore the contribution of RSA in lentil, a study with a large number of genotypes is required.

It is labour intensive and time consuming to screen a large number of genotypes under field conditions. To solve the problem, a quick phenotyping protocol was proposed in lentil for screening against drought stress hydroponically [16]. The genotypic responses in the hydroponic screening were found to be similar to the soil culture, but there are observations that the RSA in-field does not resonate with that in hydroponics [17]. Root traits were compared in lentil under drought-imposed conditions using soil and artificial media [18]. Significant variability was noticed among various root traits recorded in soil and media, and the media could not truly represent the root traits compared to the soil. Keeping this in view, the present investigation was designed with 119 lentil genotypes (93 indigenous, 24 exotic accessions and 2 varieties) using soil culture to characterise RSA in lentil germplasm. Since lentil germplasm has not been fully explored for its potential, we hypothesised that considerable genetic variation may be present in *Lens culinaris* for root traits in relation to drought stress.

## 2. Materials and Methods

Seeds of lentil germplasm (119) were procured from National GenBank, National Bureau of Plant Genetic Resources, New Delhi and Division of Genetics, Indian Agricultural Research Institute, New Delhi (Table S1). Germplasm set contained 78.15% indigenous accessions, 20.17% exotic accessions and 1.68% varieties. Selected germplasm set was not screened previously for drought tolerance.

### 2.1. Growth Conditions and Treatments

A pot experiment was carried out in randomised complete block design in four replications (five plants per replication) and two treatments during rabi season during 2018–2019 at National Bureau of Plant Genetic Resources, New Delhi (28.6331° N, 77.1525° E), India. FLIP-96-51 was used as the drought tolerant check and JL3 was used as the drought susceptible check in the study [19]. Pots (8 cm diameter) were filled with soil mixture, which had 1:1 ratio of top field soil (sandy loam, pH 7.0) and farmyard manure. Control plants were maintained at 80% field capacity and stress was imposed by maintaining the plants at 25% field capacity under controlled conditions (28/23 °C, day/night temperatures and 65% Relative humidity). Seeds of lentil genotypes (119) were treated with 1% sodium hypochlorite for 2–3 min followed by thorough washing. Seeds were germinated in dark at 22 °C in controlled conditions. After emergence, five seedlings were transferred to pots. Seedling survival (SS) was recorded visually in stressed plants on daily basis. Seedlings were harvested after 3 weeks of stress. Total dry weight (BM) was recorded by drying the seedlings (three plants per replication) in hot air oven at 70 °C for 72 h.

### 2.2. Root Traits Measurement

To record root traits, plants were uprooted and washed carefully to avoid breakage. Thereafter, shoots and roots were separated. To record different root architecture traits, roots were scanned with Epson 1680 scanner at 200 dpi and analysed using WinRHIZHO software (v9.0, Regent Instrument Inc., Quebec, QC, Canada). Root traits viz. total root length (TRL), total projected area (TPA), total surface area (TSA), average root diameter (AVD), root volume (RV), fragile root length (FRL), fragile root surface area (FRSA), fragile root volume (FRV), tips number (TN) and fork number (FN) were recorded in control as well as stressed plants. Roots with a diameter < 0.5 mm were designated as fragile roots.

Stress tolerance index (STI) was calculated for SS and BM using the formula mentioned below [20].

STI = (Yp × Ys)/(Xp)^2^ (1)

where Ys = seedling survival or biomass of a test genotype under drought stress; Yp = seedling survival or biomass of a test genotype under control condition, and Xp = mean seedling survival or biomass of test genotypes under control condition.

STI < 0.50 was considered as susceptible, 0.75 > STI < 0.50 as moderately tolerant and STI > 0.75 as highly tolerant. 

### 2.3. Statistical Analysis

Data was analysed using SPSS version 16.0 (IBM, Chicago, IL, USA) for measuring analysis of variance (ANOVA) with respect to genotype, treatments and their interactions. The least significant differences (LSD) were calculated at *p* < 0.05 and *p* < 0.01. Pearson’s correlation coefficients analysis was done to study correlations among different traits. Principal component analysis (PCA) was performed using Statistical Analysis System (SAS-JMP v14, SAS Institute, Cary, NC, USA) software to identify the contributing traits under control and drought conditions at seedling stage.

## 3. Results

We observed significant changes in root traits, survival and growth in lentil genotypes under drought. 

### 3.1. Genetic Variation in Root Traits

**Figure 1:**
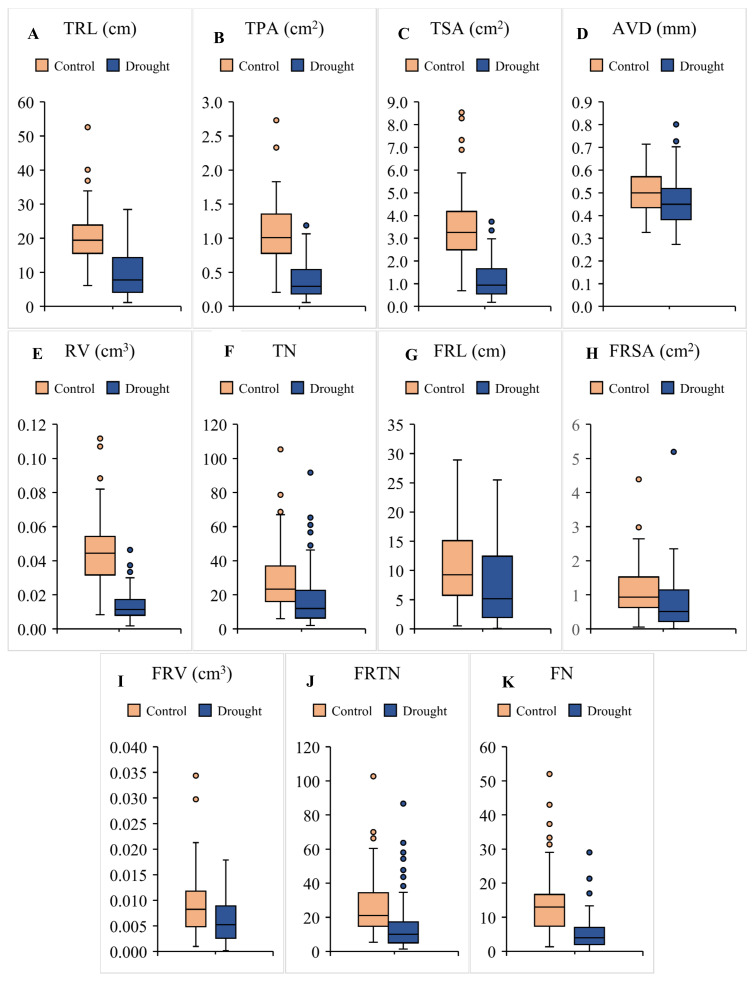
Genotypic variation in (**A**) total root length (TRL), (**B**) total projected area (TPA), (**C**) total surface area (TSA), (**D**) average diameter (AVD), (**E**) root volume (RV), (**F**) tips number (TN), (**G**) fragile root length (FRL), (**H**) fragile root surface area (FRSA), (**I**) fragile root volume (FRV), (**J**) fragile roots tips number (FRTN) and (**K**) fork number (FN) among lentil genotypes. Each box represents interquartile range. Horizontal bar in each box shows median. Whiskers show the range and dots represent outliners.

All the traits showed significant genotypic variation in control as well as stress conditions (Figure 1). Changes were significant with respect to genotype, treatment and their interactions (*p* < 0.01). The TRL reduced under stress by 54.6% among lentil lines compared to controls (Figure 1A). The root length increased or changed non-significantly in 10.9% of genotypes, while the rest decreased in TRL under a moisture-limited environment. Percent reduction in the TRL was the highest in IC559696 (94.78%), and IC559665 had the highest increment (83.4%) in response to stress.

The average TPA reduced by 70.1% in lentil lines under drought conditions (Figure 1B). Only IC559665 and IC398691 registered a significant increase; EC78511, IC559831 and IC560331 showed no significant change and rest of the lines revealed a reduction in TPA compared to controls. FLIP-96-51 showed more reduction (76.15%) compared to JL3 (64.56 %) in stress conditions. IC201701 had the least TPA (0.12 cm^2^), while IC560332 had the largest TPA (1.19 cm^2^) in water-limited conditions. The mean TSA decreased by 64.56% in the studied accessions under stress (Figure 1C). The TSA was raised in EC78391, EC78474, EC78511, IC398691, IC559665 and IC559831 in response to drought. The reduction in TSA was highest in EC78515 (94.44%), followed by IC559673 (94.43%), IC394817 (93.88%), IC208336 (92.44%) and IC559696 (92.21%). FLIP-96-51 showed less reduction (47.1%) than JL3 (77.32%) compared to controls in the water-limited condition.

Genotype-dependent variation was observed in the AVD in drought stress (Figure 1D). The AVD was augmented in 18.48% of genotypes, while 9.24% of lines showed no significant change. The rest (72.28%) accessions noted a decrease in the AVD in the water-limited environment. FLIP-96-51 and JL3 saw an 11.75% and 21.64% reduction, respectively, compared to the controls. IC424523 had the least AVD (0.33 mm) and IC248693 had the highest (0.71 mm) in control conditions, while EC78391 had the thinnest roots (0.27 mm) and IC208336 had the thickest roots (0.80 mm) in drought conditions.

The RV decreased by 69.85% in stress conditions compared to non-stress (Figure 1E). IC559665 and IC559780 registered a significant increase in RV, while no significant change was recorded in IC560032 and EC78511. Percent reduction in RV ranged 19.44–95.69% in the rest of the genotypes in response to stress. FLIP-96-51 and JL3 showed a 60.14% and 76.27% decrease in RV, respectively.

The TN reflects the extent of branching in the roots. The TN increased by 22.69% in the lentil lines, remained unchanged in IC559740 and reduction varied from 5.56% to 93.99% among the rest of the lines under stress (Figure 1F). The increase in TN was more than four times in IC559907. FLIP-96-51 showed a 31.03% reduction in TP, while JL3 showed a 71.53% decrease in the water-limited environment.

Significant variation was evident in the FRL under control as well as stress conditions (Figure 1G). The FRL increased in 22.69% of total genotypes; EC78391 had more than five times, IC559907 more than four times, EC78474, IC282829, IC361417 and IC559688 more than three times higher FRL in water-limited environments compared to the controls. In response to stress, IC559904 and IC201771 noted the maximum reductions in FRL, which were 99.23% in both the genotypes. Compared to controls, the FRL decrease was 31.84% and 54.75% in FLIP-96-51 and JL3, respectively. Like the FRL, FRSA showed significant variations under both control and stress conditions (Figure 1H). The FRSA increased in 22.69% of genotypes in response to water limitations and the genotypes followed the same pattern of change in response to stress as observed for the FRL. IC241783, IC559907, IC559740 showed reduction in the FRSA and increased in FRL under stress, compared to control. On the contrary, IC281600, IC559713, IC560032 showed increases in FRSA with a decreased FRL under stress.

Significant variation was observed for the FRV in lentil under both treatments (Figure 1I). Only 26.9% of the genotypes had increased in FRV, the other 73.1% showed reduction compared to the controls. IC266840, IC559688 and IC559769 had more than five times (and IC355621 had four times) higher FRV under water-scarce conditions. IC559904 showed the maximum decrease (99.32%) in FRV in stress conditions compared to the control conditions. The FRTN differed significantly in the control and stress conditions (Figure 1J). The FRTN reduced significantly in lentil in response to stress, though 18.4% of the genotypes increased compared to the control. Genotypes IC559907 (86.67), IC398691 (63.37) and IC267661 (60.33) showed the maximum FRTN in response to drought. FLIP-96-51 registered a 49.65% reduction and JL3 showed a 73.85% decrease in FRTN in water-limited conditions compared to controls. The FN showed significant differences in control and stress environments (Figure 1K) and mean reduction was 65.12% compared to controls. Only 10.1% of genotypes showed an increase in FN under drought conditions. EC78474 had the maximum forks (29) followed by IC385822 (21.33). FLIP-96-51 had a 30.0% and JL3 had a 34.78% decrease in FN compared to controls.

### 3.2. Growth and Survival at Seedling Stage Drought Stress

**Figure 2:**
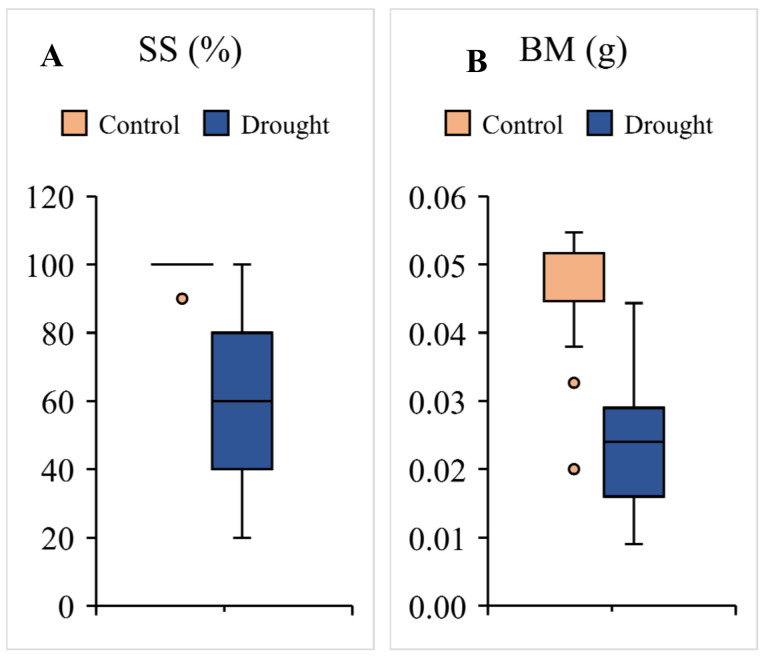
Genotypic variation for (**A**) seedling survival (SS) and (**B**) biomass (BM) among lentil genotypes. Each box represents interquartile range. Horizontal bar in each box shows median. Whiskers show the range and dots represent outliners.

Seedling survival ranged between 20% and 100% among the lentil genotypes in response to water stress (Figure 2A). Seedling survival reduced to one fifth in 8.4% of genotypes, while 20.2% of the lines had a 20–40% survival rate, followed by 40–60% in 31.1% of the lines, 60–80% in 21.8% and 80–100% in 18.5% of total genotypes. (Figure 2A). Seedling dry weight reduced remarkably under stress and the mean reduction was 50 % among the different lines. Genotype, treatment, and their interaction were significant for the trait (*p* < 0.01). The dry weight reduced to more than half in 68 lines, while, in 34 lines biomass reduced 50–75% compared to controls (Figure 2B). The dry weight reduction under stress was less than 25% in 13 lentil lines. Among 22 lines, which had a 100% seedling survival rate in the water-scarce environment, biomass reduction ranged from 12.5 to 80.0%, showing that few lines ensured their survival in response to stress, while others ensured survival along with the vigour. Drought-tolerant line FLIP-96-51 showed a 24.74% decrease in dry weight and a 90% seedling survival rate in stress. IC559673, IC559769, IC560337, IC385822, IC559713, IC560246, IC559647, IC559744, IC559696, IC560051, IC559757 and IC560032 performed better than FLIP-96-51 in terms of dry weight under stress, while seedling survival varied between 80 and 100% for all these genotypes, except for IC559673. IC559673 had a 40% survival rate under stress conditions.

### 3.3. Principal Traits and Correlation among Traits

**Figure 3:**
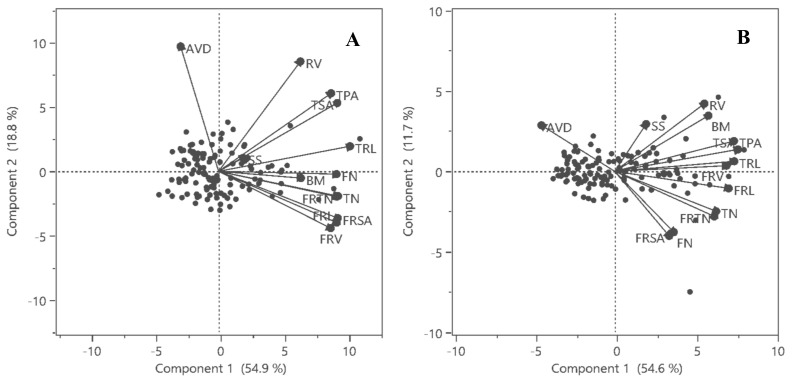
Biplot for Principal Component Analysis of root and growth traits under (**A**) control and (**B**) drought conditions.

In control conditions, PC1 and PC2 explained 73.7% of variation, while under drought, PC1 and PC2 represented 66.3% of variation (Figure 3A,B). PC1 comprised of TRL, TPA, TSA, RV, BM, TN, FRL, FRSA, FRTN in normal conditions and TRL, TPA, TSA, FRL and BM in drought conditions. PC2 was represented by the AVD in control conditions as well as in drought stress. The BM contributed more to PC1 under stress compared to controls. All the recorded traits showed a positive relationship to each other except for the AVD.

**Figure 4:**
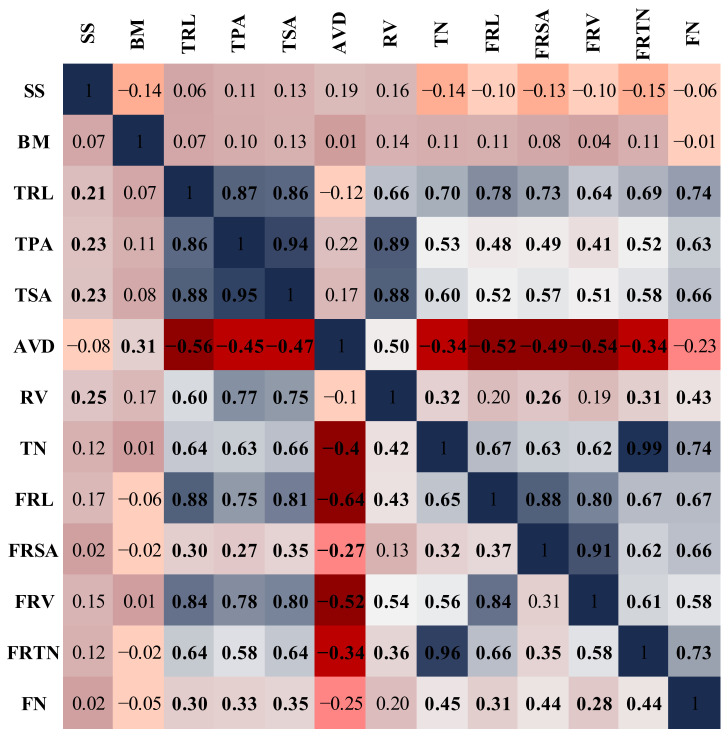
Pearson’s correlation coefficient analysis of different traits recorded under control and drought conditions at seedling stage. Values above diagonal represent control and below diagonal represent drought conditions. Colour intensity increases from Red to Blue from negative to positive correlations and values in bold show significant correlations between the traits. Seedling survival (SS), biomass (BM), total root length (TRL), total projected area (TPA), total surface area (TSA), average diameter (AVD), root volume (RV), tips number (TN), fragile root length (FRL), fragile root surface area (FRSA), fragile root volume (FRV), fragile roots tips number (FRTN), fork number (FN).

Correlation analysis revealed significant positive correlations among different root traits under control and drought conditions (Figure 4). In the control condition, the BM and SS were not correlated to any root trait. The TRL and TPA had a positive relationship to all other root traits except for the AVD in control, while the TSA revealed a positive relationship to all root traits. Significant positive correlations were recorded between the RV and AVD (r = 0.50), TN (r = 0.32), FRSA (r = 0.26), FRTN (r = 0.31) and FN (r = 0.43). The AVD showed a negative relationship to the TN (r = −0.34), FRL (r = −0.52), FRSA (r = −0.49), FRV (r = −0.54) and FRTN (r = −0.34). The FRL, FRSA, FRV, FRTN and FN shared strong positive correlations to all the root traits except for the AVD. The SS was positively correlated to the TRL (r = 0.21), TPA (r = 0.23), TSA (r = 0.23) and RV (r = 0.25), while the BM showed negative correlations to the AVD (r = −0.31) under stress conditions. The TRL showed positive correlations to all other root traits except for the AVD (r = −0.56), where it had a significant negative relationship. The TPA, TSA, RV, TN, FRL, FRSA, FRV, FRTN and FN all showed a positive relationship to each other, except for the AVD.

### 3.4. Selection of Drought Tolerant Genotypes at Seedling Stage

**Figure 5:**
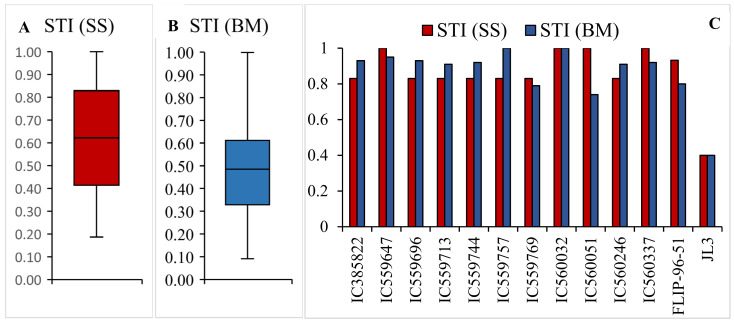
Box plot representation of (**A**) STI (SS), (**B**) STI (BM) in lentil germplasm and (**C**) bar diagram for STI(SS) and STI (BM) in drought-tolerant lentil lines.

The STI based on SS ranged from 0.19 to 1.0 among the lentil germplasm (Figure 5A). In total, 27.77% of genotypes reported STI < 0.50, while 31.93% of the lines showed an STI between 0.50 and 0.75 and 40.3% of the lines had STI > 0.75. When the STI was calculated based on the dry weight, it ranged between 0.09 and 0.900 (Figure 5B). The STI was less than 0.50 in 51.10% of lentil lines, while 31.25% of the lines had an STI between 0.50 and 0.75. The STI was greater than 0.75 in 17.65% of total lentil germplasm lines. A total of 11 genotypes exhibited a STI (SS) and STI (DW) greater than 0.75 and these selected genotypes were classified as drought-tolerant. Identified genotypes are IC559647, IC560051, IC560032, IC560037, IC560246, IC559769, IC559757, IC559744, IC559713, IC559696 and IC385822 (Figure 5C).

## 4. Discussion

The root is the prime organ involved in water uptake. The contribution of root traits becomes more critical in water-limited environments, extracting water from deeper layers. The above-ground traits have always gained the attention of breeders, while the root traits have often been neglected due to their complex phenotyping and low heritability [21]. Over the past decade, different studies have focused on phenotyping and breeding for root traits to improve drought tolerance using genomic, transcriptomic and metabolomic approaches [22,23,24,25,26]. Though the studies have been carried out in chickpea [12], common bean [11] and other legumes, RSA in lentil under drought has not been analysed to date. Therefore, we analysed the RSA in lentil under normal and drought conditions.

The present study revealed significant variations for all the recorded root traits with regard to genotype, treatment and their interactions at seedling stage, under control and low moisture conditions (Figure 1, Table S2). The TRL reduced significantly on exposure to stress, but the extent of the reduction was genotype-dependent. The TRL was significantly correlated to survival at seedling stage. The TRL reflects the ability of a plant to exploit moisture in water-scarce conditions, we observed that the genotypes with a higher TRL were better in survival and growth under water deficiency conditions. Similar observations were recorded in soybean [27], oats [28] and chickpea [12].

The root surface area represents the proportion of root interacting with the soil profile. Besides this, the TSA, AVD and RV also determine the ability of plants to access water and nutrients [27,29]. The TPA and TSA increased in FLIP-96-51 and IC559696 in response to the water-limited environment at seedling stage. All the selected genotypes responded differently to drought with regard to the TSA, but the TPA and TSA were positively correlated to the BM under drought conditions. It was revealed that the trait contributed significantly to water extraction in water-limited conditions. Likewise in soybean, the seedling stage drought led to an increase in the TRL, TSA and RV, while drought at other stages revealed a reduction in root growth [30]. The AVD shows the thickness of roots. It is well established that roots with a higher AVD are more efficient in water uptake, while roots with a lower AVD contribute more towards increasing the surface area [31,32]. We observed reduction in root diameter in all the selected lines. The AVD also showed significant negative correlations to the BM under stress. This bespeaks the importance of traits in lentil with regard to drought tolerance. We observed that thin roots contributed significantly to water uptake in drought-tolerant lines. Contrarily, positive correlations were recorded between the yield and metaxylem plasticity and xylem diameter in soybean under drought stress [33].

FLIP-96-51 showed a decrease in RV, while IC560051 registered no reduction in drought. The TN fell in FLIP-96-51 and JL3 at seedling stage, but the trait showed no significant correlation to the SS or BM under drought conditions. Root-branching represented by the TN shows the tendency of the different genotypes to explore different soil layers in order to access water. Fine root traits (root diameter < 0.5 mm) (FRL, FRSA, FRV, FRTN and FN) in lentil germplasm reduced in response to the water deficit (Figure 1). Genotypic variation was significant in all the genotypes, irrespective of their response towards drought. The study showed that these traits changed under stress, but were not correlated to growth in stress. On the contrary, the role of the fine root at different soil depths was reported in deciding their response towards water scarcity in cultivated and wild lentil [34]. The TRL, TSA, AVD, PRL and RV were identified as the most contributing traits in mung bean core-collection at seedling level [35].

Seedling survival and biomass varied remarkably in response to stress among the genotypes (Figure 2; Table S2, Figures S1 and S2). Drought-tolerant check FLIP-96-51 reported 90% SS along with 24.74% reduction in dry weight compared to non-stress. Our results were in accordance with a previous study in lentil, where QTLs associated to SS were identified in RIL populations [16,19]. The SS and drought score based on visual stress injury symptoms were used as the selection criterion for drought under hydroponics and germination, relative water content and RL in PEG mediated stress in lentil [16,36].

The PCA and correlation studies showed that the TRL, TPA, TSA, RV and AVD were the major root traits responsible for variation under the control and drought conditions (Figure 3 and Figure 4). Most of the recorded root traits were positively associated with SS and only the AVD had a negative relationship to BM (Figure 4). This shows that a thin root played a more active role in the growth compared to other root traits which were significant for survival. However, we did not observe a significant association between other fine-root traits and BM at seedling stage. We observed that thinner and longer roots with more surface area were instrumental against drought stress. Our results complied with the observations in wheat [37] and common bean [38], where the RSA at seedling stage was correlated to drought tolerance in field.

In conclusion, the study revealed that the RSA played an important role in survival and growth of lentil under drought conditions. We identified drought tolerant lentil genotypes (IC560051, IC560246, IC559769, IC559757, IC559744, IC559713, IC559696 and IC835822, IC559647, IC560032, and IC560337) which can be utilised as trait donors in a lentil breeding program.

## Data Availability

The data that supports the findings of this study are available in the Supplementary Material of this article.

## Supplementary Materials

The following are available online at https://www.mdpi.com/article/10.3390/plants10112410/s1, Figure S1: Percent seedling survival (SS) of studied lentil genotypes under control and drought conditions, Figure S2: Biomass (BM) of studied lentil genotypes under control and drought conditions, Figure S3: WinRHIZHO root imaging system (left) and representative root image (right) of lentil genotype; Table S1: List of lentil germplasm screened for drought tolerance at seedling stage, Table S2: Mean and range for recorded traits in lentil germplasm under control and drought conditions.
