# Supplementary material for: Root Trait Variation in Lentil (Lens culinaris Medikus) Germplasm under Drought Stress
Source: Plants (Basel). 2021 Nov 9;10(11):2410. doi: 10.3390/plants10112410 (PMC8621538; doi:10.3390/plants10112410)
Supplement: Supplementary file 1 [file plants-10-02410-s001.zip › plants-1392443-supplementary figures.pdf]

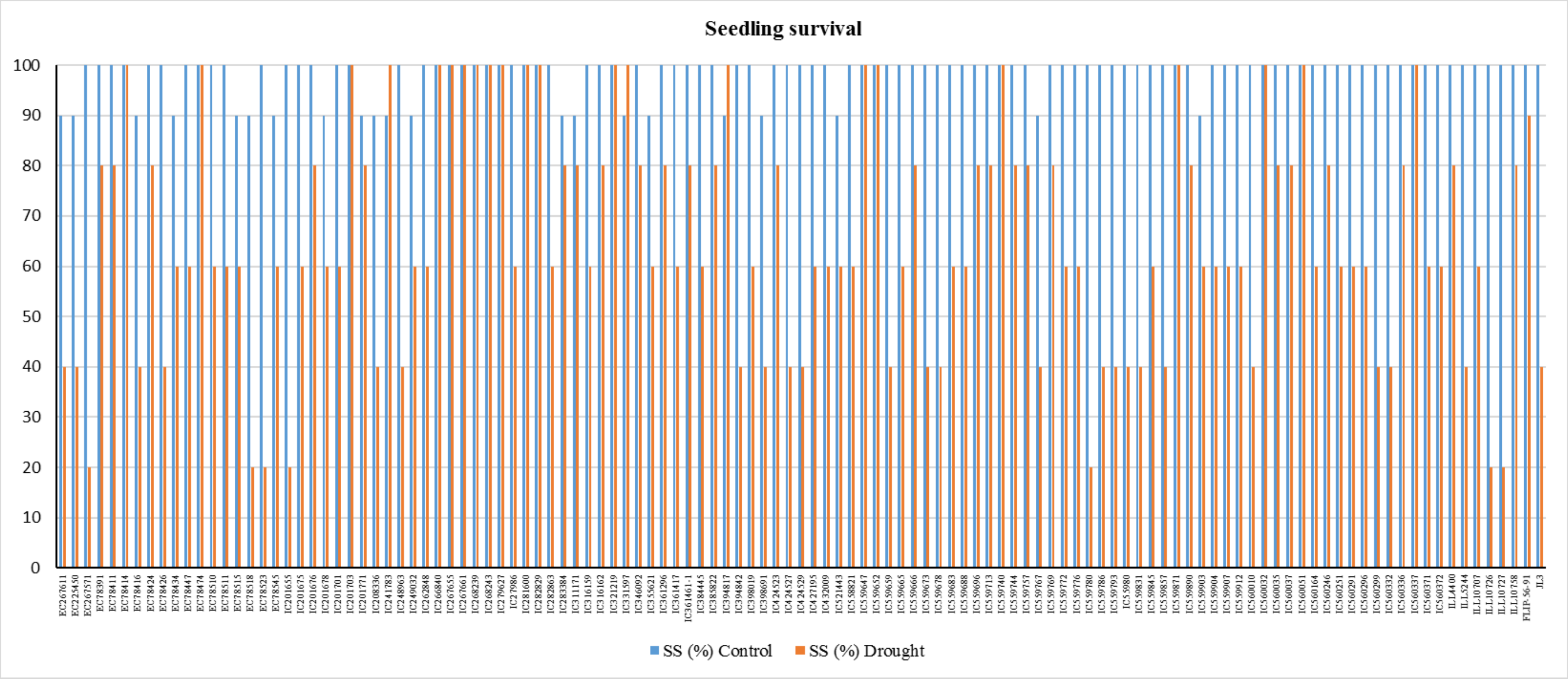

Figure S1. Percent seedling survival (SS) of studied lentil genotypes under control and drought conditions

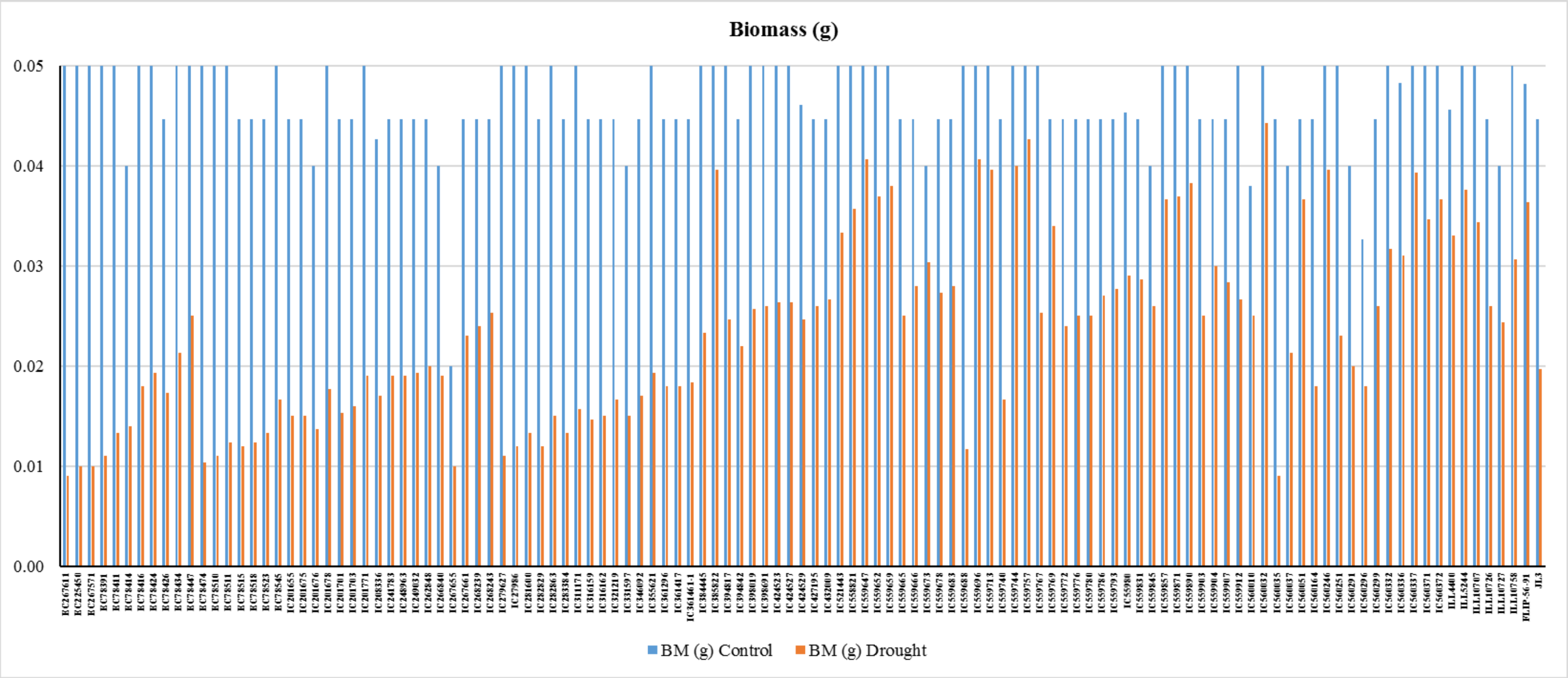

Figure S2 Biomass (BM) of studied lentil genotypes under control and drought conditions

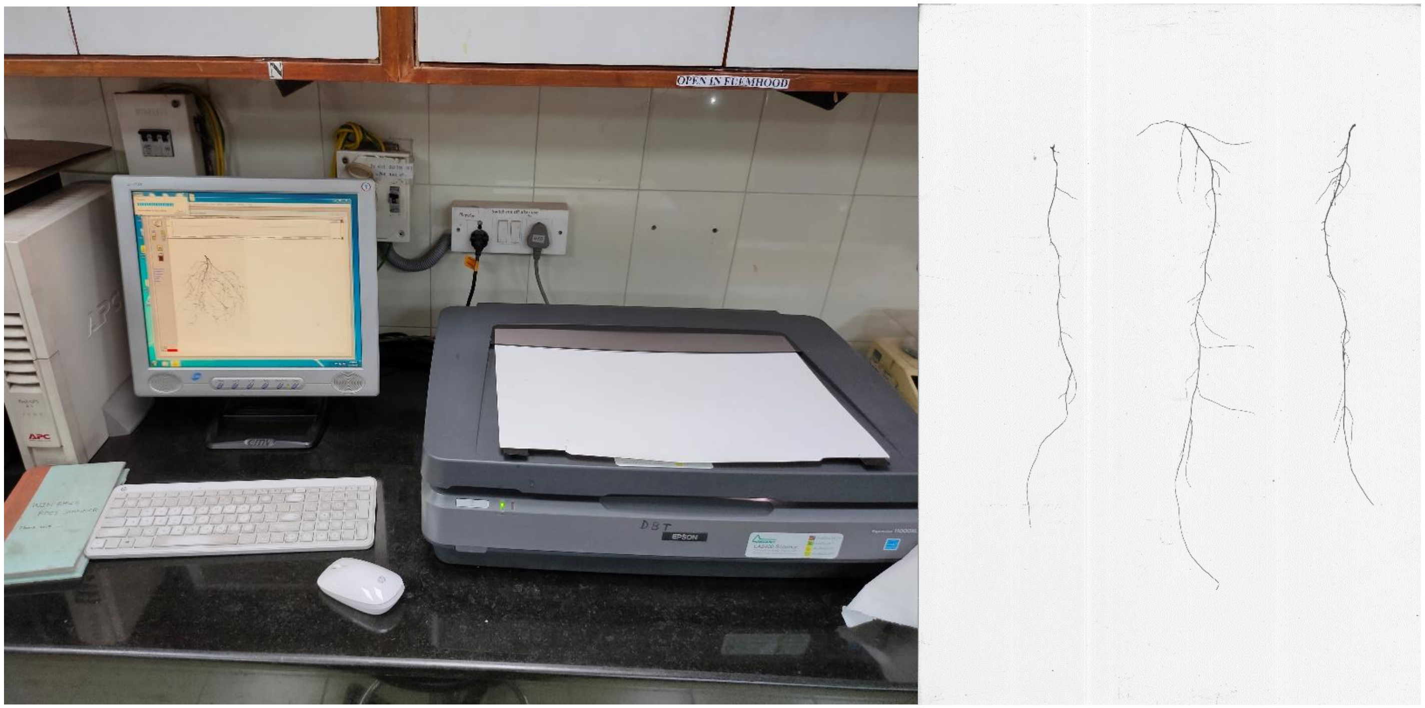

Figure S3 WinRHIZHO root imaging system (left) and representative root image (right) of lentil genotype
