# Supplementary material for: Root Trait Variation in Lentil (Lens culinaris Medikus) Germplasm under Drought Stress
Source: Plants (Basel). 2021 Nov 9;10(11):2410. doi: 10.3390/plants10112410 (PMC8621538; doi:10.3390/plants10112410)
Supplement: Supplementary file 1 [file plants-10-02410-s001.zip › plants-1392443-supplementary Tables.pdf]

Table S1. List of lentil germplasm screened for drought tolerance at seedling stage

**S. N.**	**Accession ID**	**Type**	**S. N.**	**Accession ID**	**Type**	**S. N.**	**Accession ID**	**Type**
1	EC267611	Exotic	49	IC361296	Indigenous	97	IC560032	Indigenous
2	EC225450	Exotic	50	IC361417	Indigenous	98	IC560035	Indigenous
3	EC267571	Exotic	51	IC361461	Indigenous	99	IC560037	Indigenous
4	EC78391	Exotic	52	IC384445	Indigenous	100	IC560051	Indigenous
5	EC78411	Exotic	53	IC385822	Indigenous	101	IC560164	Indigenous
6	EC78414	Exotic	54	IC394817	Indigenous	102	IC560246	Indigenous
7	EC78416	Exotic	55	IC394842	Indigenous	103	IC560251	Indigenous
8	EC78424	Exotic	56	IC398019	Indigenous	104	IC560291	Indigenous
9	EC78426	Exotic	57	IC398691	Indigenous	105	IC560296	Indigenous
10	EC78434	Exotic	58	IC424523	Indigenous	106	IC560299	Indigenous
11	EC78447	Exotic	59	IC424527	Indigenous	107	IC560332	Indigenous
12	EC78474	Exotic	60	IC424529	Indigenous	108	IC560336	Indigenous
13	EC78510	Exotic	61	IC427195	Indigenous	109	IC560337	Indigenous
14	EC78511	Exotic	62	IC432009	Indigenous	110	IC560371	Indigenous
15	EC78515	Exotic	63	IC521443	Indigenous	111	IC560372	Indigenous
16	EC78518	Exotic	64	IC558821	Indigenous	112	ILL4400	Exotic
17	EC78523	Exotic	65	IC559647	Indigenous	113	ILL5244	Exotic
18	EC78545	Exotic	66	IC559652	Indigenous	114	ILL10707	Exotic
19	IC201655	Indigenous	67	IC559659	Indigenous	115	ILL10726	Exotic
20	IC201675	Indigenous	68	IC559665	Indigenous	116	ILL10727	Exotic
21	IC201676	Indigenous	69	IC559666	Indigenous	117	ILL10758	Exotic
22	IC201678	Indigenous	70	IC559673	Indigenous	118	FLIP-56-91	Variety
23	IC201701	Indigenous	71	IC559678	Indigenous	119	JL3	Variety
24	IC201703	Indigenous	72	IC559683	Indigenous			
25	IC201771	Indigenous	73	IC559688	Indigenous			
26	IC208336	Indigenous	74	IC559696	Indigenous			
27	IC241783	Indigenous	75	IC559713	Indigenous			
28	IC248963	Indigenous	76	IC559740	Indigenous			
29	IC249032	Indigenous	77	IC559744	Indigenous			
30	IC262848	Indigenous	78	IC559757	Indigenous			
31	IC266840	Indigenous	79	IC559767	Indigenous			
32	IC267655	Indigenous	80	IC559769	Indigenous			
33	IC267661	Indigenous	81	IC559772	Indigenous			
34	IC268239	Indigenous	82	IC559776	Indigenous			
35	IC268243	Indigenous	83	IC559780	Indigenous			
36	IC279627	Indigenous	84	IC559786	Indigenous			
37	IC27986	Indigenous	85	IC559793	Indigenous			
38	IC281600	Indigenous	86	IC55980	Indigenous			
39	IC282829	Indigenous	87	IC559831	Indigenous			
40	IC282863	Indigenous	88	IC559845	Indigenous			
41	IC283384	Indigenous	89	IC559857	Indigenous			
42	IC311171	Indigenous	90	IC559871	Indigenous			
43	IC316159	Indigenous	91	IC559890	Indigenous			
44	IC316162	Indigenous	92	IC559903	Indigenous			
45	IC321219	Indigenous	93	IC559904	Indigenous			
46	IC331597	Indigenous	94	IC559907	Indigenous			
47	IC346092	Indigenous	95	IC559912	Indigenous			
48	IC355621	Indigenous	96	IC560010	Indigenous			

Table S2. Mean and range for recorded traits in lentil germplasm under control and drought conditions

Traits	Range		Mean	
	Control	Drought	Control	Drought
Total root length (cm)	6.13-52.58	1.16-28.46	20.65	9.38
Total projected area (cm^2)	0.21-2.73	0.06-1.19	1.05	0.37
Total surface area (cm^2)	0.69-8.53	0.19-3.73	3.39	1.17
Average diameter (cm)	0.33-0.71	0.27-0.80	0.51	0.46
Root volume (cm^3)	0.008-0.112	0.002-0.046	0.04	0.01
Tips number	6.00-105.33	2.00-91.67	27.44	17.06
Fragile root length (cm)	0.54-28.90	0.08-25.50	10.84	7.03
Fragile root surface area (cm^2)	0.06-4.39	0.001-5.24	1.12	0.70
Fragile root volume (cm^3)	0.001-0.034	0.0001-0.0179	0.009	0.006
Fragile root tips number	5.33-102.67	1.33-86.67	25.62	14.50
Fork number	1.33-52.00	0.00-29.00	13.93	4.82
Biomass (g)	0.020-0.055	0.009-0.044	0.047	0.024
Seedling survival (%)	90-100	20-100	98.24	65.63
